# Editorials

**Published:** 1910-10

**Authors:** 


### EDITORIALS

The Business Office of the *JOURNAL-RECORD* is 1 1-2 to 5 1-2 South Broad Street.
The Editorial Office is 1014-15 Century Building.
Address all Business Communications to *Journal-Record of Medicine*, 1 1-2 to 5 1-2 South Broad Street.
Make remittances payable to *THE JOURNAL-RECORD OF MEDICINE.*
On matters pertaining to the Editorial and Original Communications, address Edgar G. Ballenger, M. D., Atlanta, Ga.
Reprints of Original Articles will be furnished at cost price. Requests for the same should always be made in THE MANUSCRIPT.
We will present, postpaid, on request, to each contributor of an original article, twenty (20) marked copies of *THE JOURNAL-RECORD OF MEDICINE* containing such article.

---

### WHY GEORGIA SHOULD ADOPT THE MEDICAL COMMISSION PLAN.

The crying defect in the present method of commitment in our State is the fact that commitment has to be in open court by requisition before a jury. This is a source of great humiliation and hardship both to the patient and the relatives. It places the unfortunate afflicted with insanity in the same category as one accused of crime.

Attend if you will, one of the hearings of our commitment proceedings, and you will be impressed both by the method of procedure and the terminology employed, that the alleged insane is tried legally as the criminal.

When we realize that insane people are sick, it is then that the commission plan is apparent. Modern civilization stands appaled at the idea that a sick woman should be tried in public before a jury, by a jury, of whom all are laymen except one, after the mahner of a thief or a murderer—before she can avail herself of the quiet and medical treatment which is indicated in all cases.

Doctors now realize the great value of early scientific treatment of incipient and acute cases of insanity.

As our law stands today, nothing appears to indicate that the unfortunate insane person is committed to the institution for his welfare, to be treated for disease, and, if possible, restored to sanity and useful citizenship,—the implication being, that he is removed from the community because he is a dangerous element.

Dr. King's idea as set forth in his paper, and the bill that he and his committee have drawn, is to make the State Sanitarium of Georgia easy of access to the afflicted, to do away with a method that brings shame and humiliation upon the patient and the patient's friends and relatives, by frightening and disturbing the patient to such an extent that the recovery is often interfered with. As a step to a more humane recognition of insanity, we believe the Medical Commission plan is the needed substitute for the rigors of a jury trial.

The legal decision of all cases of insanity is based on Medical opinion, whether proceedings be by jury or commission. The question of insanity is solely a medical question to be solved by medical men. The law-makers of our State should realize that if a man or woman is sick in any other organ of the body except the brain or nervous system, a court would be the last place on earth to think of taking such a person.

Considering that only seven states in the Union have commitment of its insane by jury, that the greatest per cent of insane cases yields to scientific treatment, as much so as other diseases, if taken early in hand and treated, realizing that insanity is a disease and not a crime, we earnestly advocate the ideas as set forth in Dr. King's paper, which appears in this issue of the Journal and Record.

---

Dr. James Preston Watkins, of Opelika, Alabama, died at his home in that city, August 18th, after a three months illness of Nephritis.

He was born at Camp Hill, Alabama, 1869. Graduated at the Southern University at Greensboro, Alabama, 1891, after which he begun the study of Medicine, graduating from the College of Physicians and Surgeons, Baltimore, Md., in 1897.

At the time of his death he was a member of the American Medical Association, Alabama State Medical Association, Lee

County Medical Society and the Chattahoochee Valley Medical and surgical Association. The last of which he did much work of value in building it up, and was President of it's Board of Council at th time of his death.

Dr. Watkins was a man of highest moral character, and of pleasing personality. He possessed remarkable clinical abilities, and his friends were those who knew him.

The los of so fine a man will be keenly felt by his many friends both in and out of the profession, and the deepest sympathy is felt throughout this section for his brothers and sisters intheir great sorrow.

